# Analysis of the influence of advanced maternal age on gestational and fetal outcomes

**DOI:** 10.61622/rbgo/2025rbgo91

**Published:** 2025-11-18

**Authors:** Rômulo Felipe Auler, Ramon Henrique Auler, Ana Clara Machado Teixeira, Giovanna Jost Tibolla, Vinícius Pessoa Nunes Vieira, Junior Bolsonelo, Edimárlei Gonsales Valério, Edison Capp

**Affiliations:** 1 Universidade Federal do Rio Grande do Sul Porto Alegre RS Brazil Universidade Federal do Rio Grande do Sul, Porto Alegre, RS, Brazil.; 2 Hospital de Clínicas de Porto Alegre Porto Alegre RS Brazil Hospital de Clínicas de Porto Alegre, Porto Alegre, RS, Brazil.; 3 Universidade Federal do Rio Grande do Sul Faculdade de Medicina Departamento de Ginecologia e Obstetrícia Porto Alegre RS Brazil Departamento de Ginecologia e Obstetrícia, Faculdade de Medicina, Universidade Federal do Rio Grande do Sul, Porto Alegre, RS, Brazil.

**Keywords:** Maternal age, Pregnant woman, Pregnancy outcome, Prenatal care, Premature birth

## Abstract

**Objective:**

Advanced maternal age has become more common due to factors such as increased academic preparation for women, labor market participation, delayed family planning, and advances in reproductive medicine. This phenomenon raises questions about the risks and gestational outcomes associated with advanced maternal age (AMA). This study aims to explore the gestational and perinatal outcomes in pregnant women with AMA (> 35 years), comparing them with those of women aged 20 to 34 years.

**Methods:**

The research was conducted as an observational and retrospective analysis, examining data from 2012 to 2022 from the Hospital Production System (SIH/SUS) available on the DATAsus platform, covering all regions of Brazil. Variables analyzed included gestational duration, type of pregnancy (single or multiple), mode of delivery, Robson group, APGAR score at five minutes, birth weight, and occurrence of congenital anomalies.

**Results:**

Pregnant women with AMA showed statistically significant differences in the higher percentage of cesarean sections (63.3% AMA vs 58% non-AMA, p<0.05), higher percentage of low birth weight newborns (12.5% AMA vs 6.7% non-AMA, p<0.05), lower APGAR scores at the fifth minute (OR = 1.08, p<0.001) and a higher prevalence of any congenital anomalies (1.23% AMA vs 0.77% non-AMA, OR =OR = 1.34, p < 0.001). In addition, this group had a higher incidence of premature births (12.99% AMA vs 8.78% non-AMA) and multiple pregnancies (1.39% AMA vs 0.83% non-AMA). Furthermore, in the Robson classification, there was a predominance of older mothers in Group 5 (previous cesarean section) and in the groups with nulliparity.

**Conclusion:**

Pregnant women with AMA face higher gestational and perinatal risks, such as preterm births and an increased need for cesarean sections. These findings underscore the importance of public policies and personalized management strategies to improve maternal and neonatal outcomes for older pregnant women.

## Introduction

In recent decades, advanced maternal age (AMA) has become an increasingly common phenomenon in contemporary society. The rise in women's academic preparation and the consequent pursuit of new professional goals, along with active participation in the labor market, have contributed to delayed family planning. Additionally, the widespread use of contraceptive methods, the emphasis on family and financial planning, and advances in reproductive medicine, which enable pregnancies at older ages, further support this reality.

Thus, there is a growing interest in investigating how AMA can influence gestational factors. This study aims to explore the medical implications of late motherhood. Furthermore, in low- and middle-income countries (LMIC), such as Brazil, the implications of AMA must be interpreted within the context of limited access to prenatal care and higher prevalence of unmanaged comorbidities, as highlighted by the World Health Organization (WHO) reports and supported by UpToDate guidelines ("Prenatal care in low- and middle-income countries", updated 2024). Among these comorbidities, diabetes mellitus gestational and hypertensive disorders, notably preeclampsia, are particularly relevant, as they significantly increase maternal and perinatal risks associated with AMA (UpToDate, "Advanced maternal age and pregnancy", updated 2024).

Regarding the Robson Classification, it was developed in 2001 by Dr. Michael Robson to monitor and categorize cesarean sections.^(1)^ A systematic review by Torloni et al.,^(2)^ which analyzed various cesarean classification systems, concluded that the Robson Classification is the most suitable tool to meet local and international needs. This system enables healthcare professionals to identify specific groups of pregnant women who may be more susceptible to complications, facilitating the implementation of personalized management strategies and preventive interventions. This not only improves maternal and neonatal outcomes but also optimizes the use of medical resources, making the Robson Classification an essential tool in modern obstetrics, particularly in the context of advanced maternal age.

The choice between vaginal delivery and cesarean section in each maternal age group aims to determine whether maternal age is indeed a determining factor in the decision-making process for the type of delivery in Brazil. This choice often serves as an indicator of gestational complications and/or maternal medical conditions that make vaginal delivery risky. Therefore, understanding the prevalence of each type of delivery across different maternal age groups is vital to infer the occurrence of complications associated with AMA.

The APGAR (Appearance, Pulse, Grimace, Activity, and Respiration) score is assessed at one and five minutes of life, and repeated every five minutes if the score remains below 7 at the fifth minute. Low APGAR scores at five minutes are associated with increased mortality and a higher population risk of cerebral palsy. A meta-analysis by Pinheiro et al.^(4)^ found an association between advanced maternal age (AMA) and APGAR scores <7 at five minutes, with odds ratios ranging from 1.09 to 1.17.^(3,4)^

Abnormal birth weight significantly increases the risk of perinatal morbidity and mortality. In recent years, it has been recognized as a risk marker for maternal age-related conditions. Additionally, it is estimated that the prevalence of low birth weight (LBW) ranges from approximately 5-7% in developed countries to as high as 19% in developing countries. This disparity further emphasizes the need for targeted studies in LMIC contexts.^(5)^

Another outcome evaluated in the literature is the presence or absence of congenital anomalies and their prevalence across different maternal age groups. During intrauterine development, structural or functional alterations can occur, affecting various organs and systems. These are caused by genetic, infectious, nutritional, and environmental factors, often resulting from a combination of these influences, including maternal age. Therefore, it is essential to investigate whether there is a direct relationship between the presence and type of congenital anomalies and pregnancies in advanced maternal age.

This study aims to evaluate gestational and perinatal outcomes in women with advanced maternal age compared to younger mothers.

## Methods

A retrospective observational study was conducted, collecting all available data between 2012 and 2022 on gestational and perinatal outcomes, which include pregnant women aged from under 10 to 69 years. These data were obtained through Tabnet, part of the Hospital Information System (SIH/SUS), available on the DATASUS platform, and cover all regions of Brazil.

In our study, advanced maternal age (AMA) was defined as women aged 35 years or older at the time of delivery (Group 1), while the age group of 20 to 34 years was considered the control group, representing non-advanced maternal age (Group 2). Pregnant women aged 19 years or younger—despite not being the focus of the study and comparative analyses due to specific clinical and legal considerations inherent to this age range—were included in the tables to ensure data transparency and highlight the quantitative impact of this group. The final analyzed sample consisted of 4,494,761 live births from mothers with AMA, out of a total of approximately 31 million registered births in Brazil between 2012 and 2022.

The variables analyzed included: gestational duration (in weeks), type of pregnancy (singleton or multiple), mode of delivery, Robson classification, Apgar score at five minutes, and the occurrence and classification of congenital anomalies in newborns.

For statistical analysis, chi-square tests were applied to assess associations between categorical variables, while logistic regression models were used to estimate the strength of associations between maternal age groups and outcomes of interest. Proportions of each outcome type were calculated relative to the absolute number of live births (LB) within each maternal age group for each variable. A p-value <0.05 was considered statistically significant.

For the Robson classification and types of congenital anomalies, data were grouped into the following age ranges: less than 20 years, 20 to 34 years (Group 2), and 35 years or older (Group 1). This grouping method was chosen based on the distribution of results, as there was minimal variation among subgroups within the same category.

Gestational age was divided into seven groups (<22 weeks, 22-27 weeks, 28-31 weeks, 32-36 weeks, 37-41 weeks, ≥42 weeks, and unknown). Pregnancy type was categorized into four groups (single, twin, triplet or higher, and unknown). For mode of delivery, three groups were defined (vaginal, cesarean, and unknown). For birth weight, LBW was defined as <2,500 grams, and macrosomia as ≥4,000 grams. The Robson Classification categorizes pregnancies into ten groups based on five obstetric characteristics:

1. Parity (nulliparous or multiparous);
2. Gestational age (term or preterm);
3. Number of fetuses (single or multiple);
4. Fetal presentation (cephalic, breech, or transverse);
5. Onset of labor (spontaneous, induced, or elective cesarean without labor).^(1)^

The ten Robson groups are:

- Group 1: Nulliparous women with a single, cephalic fetus at ≥37 weeks in spontaneous labor.
- Group 2: Nulliparous women with a single, cephalic fetus at ≥37 weeks undergoing induced labor or elective cesarean before labor onset.
- Group 3: Multiparous women without previous cesarean, with a single, cephalic fetus at ≥37 weeks in spontaneous labor.
- Group 4: Multiparous women without previous cesarean, with a single, cephalic fetus at ≥37 weeks undergoing induced labor or elective cesarean before labor onset.
- Group 5: Multiparous women with at least one previous cesarean, with a single, cephalic fetus at ≥37 weeks.
- Group 6: Nulliparous women with a single fetus in breech presentation.
- Group 7: Multiparous women with a single fetus in breech presentation, including those with previous cesareans.
- Group 8: Women with multiple gestations, including those with previous cesareans.
- Group 9: Women with a fetus in transverse or oblique presentation, including those with previous cesareans.
- Group 10: Women with a single, cephalic fetus at <37 weeks, including those with previous cesareans.^(6)^

For outcome analysis, APGAR scores were categorized into: scores < 8 and scores 8-10. Maternal age was also stratified into Group 1 (AMA): ≥35 years and Group 2 (control group): 20-34 years. We chose to group APGAR scores into two categories because Pinheiro et al.^(4)^ used this same stratification (Table 3). (Note: DataSUS provides the data already stratified by different age groups).

Congenital anomaly rates were adjusted according to the number of live births within each maternal age range to identify findings. Fetal mortality outcomes were not evaluated in this study. For the variable assessing the presence or absence of anomalies, the data were detailed for Group 1, while Group 2 was presented in aggregate form.

Regarding missing data, variables with missing or unknown entries were maintained as separate categories, following the guidelines of the public databases (DATASus), ensuring transparency in data presentation and analysis.

## Results

**Figure 1:**
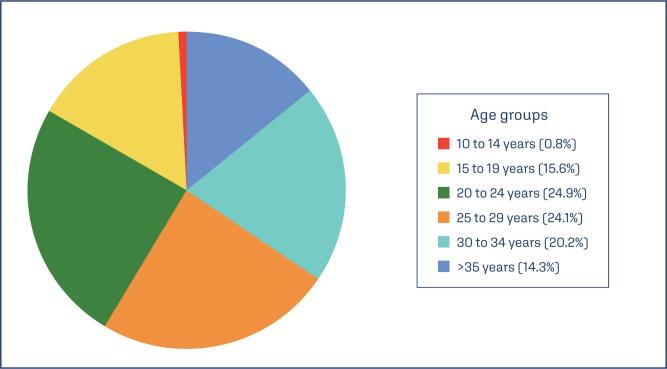
Distribution of age groups

During the evaluated period, from 2012 to 2022, there were 4,494,761 live births from women with advanced maternal age (AMA), accounting for approximately 14.3% of all births, according to DATASus data (Figure 1).

**Figure 2:**
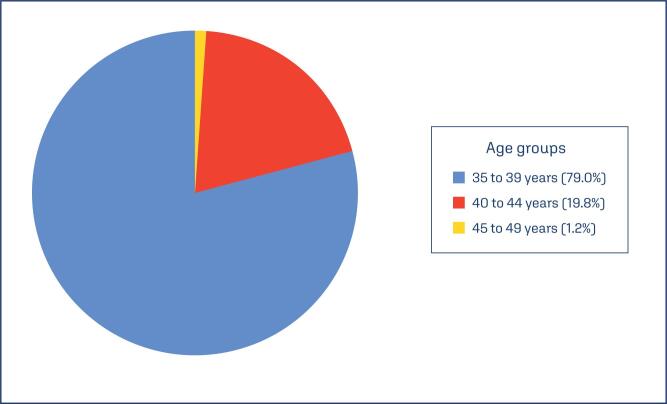
Distribution of advanced maternal age groups

Among pregnancies at an advanced age, the majority occurred within the 35 to 39 age range (79%), followed by the 40 to 44 and 45 to 49 ranges, with 19.8% and 1.2%, respectively (Figure 2).

Below, the results are presented separately for each variable analyzed based on DATASus data, adjusted by maternal age group. It is important to highlight that the tables present data for all maternal age groups available on the DATASus platform, aiming to ensure data transparency and to show values for all groups. However, for analytical and comparative purposes, the age groups of 20–34 years (non-AMA) were considered in comparison to those aged 35 and over (AMA). Regarding gestational age, the highest number of births (over 70%) occurred between 37 and 41 weeks across all age groups. Nevertheless, slight variations were observed. Among women aged 20 to 34, the prevalence of preterm births (32-36 weeks) ranged from 8.71% to 9.70%, whereas for women aged 35 and above, preterm birth rates were higher, ranging from 11.23% to 18.79%, with the highest rate observed in the 50-54 age group (Table 1). Women ≤19 years had a higher proportion of extreme prematurity (<22 weeks) and late prematurity (32-36 weeks) compared to the 20-34 years group. Women ≥35 years had a higher proportion of moderate prematurity (28-31 weeks) and late prematurity (32-36 weeks) compared to the 20-34 years group.

**Table 1:** Gestational age in weeks

Gestational age	≤19 years (%)	20-34 years (%)	≥35 years (%)	Unknown age (%)	Total	p-value
<22 weeks	0.08	0.05	0.04	0.15	15,915	<0.001
22-27 weeks	0.66	0.45	0.53	0.29	155,851	<0.001
28-31 weeks	1.28	0.92	1.23	1.62	321,26	<0.001
32-36 weeks	10.78	9.02	11.65	11.21	3,037,542	<0.001
37-41 weeks	80.69	84.69	83.09	56.64	26,274,041	<0.001
≥42 weeks	3.64	2.73	1.68	5.90	855,948	<0.001
Unknown Age	2.87	2.14	1.76	24.19	690,767	<0.001
Total	5,149,998	21,705,887	4,494,761	678,031	31,351,324	

Regarding the number of fetuses per pregnancy, single gestations predominated among women aged 20 to 34, accounting for 97.75% to 98.24% of births, followed by twin pregnancies, ranging from 1.59% to 2.68%. For women aged 35 and above, single pregnancies remained predominant, ranging from 86.32% to 100%, followed by twin pregnancies (0.00%-12.80%). In the 45-49, 50-54, and 55-59 age groups, the proportion of twin pregnancies was significantly higher than in other age ranges, with respective rates of 5.41%, 12.80%, and 7.77%. In the Robson Classification, women with AMA predominantly fell into Group 5 (with a previous cesarean section, single, cephalic fetus, >37 weeks), representing 28.96%. Regarding nulliparity, when combining Groups 1 (nulliparous, single, cephalic fetus, >37 weeks in spontaneous labor), 2 (nulliparous, single, cephalic fetus, >37 weeks with induced labor or pre-labor cesarean), and 6 (all breech deliveries in nulliparous women), the proportion in AMA (12.26%) was nearly half compared to women aged 20 to 34 (24.82%). A nearly twofold increase was observed in AMA women in Group 9 (all pregnancies with fetuses in transverse or oblique presentation, including previous cesareans), with 0.28% compared to 0.16% in the 20-34 group. Group 10 (single, cephalic fetuses, <37 weeks) also showed a slight increase in AMA women (8.71%) compared to the control group (6.79%), indicating a higher risk of preterm birth in older mothers (Table 2). Women ≥35 years had a higher probability of being in groups 3-5 (OR = 1.19) and 6-7 (OR = 1.15) compared to the 20-34 years group.

**Table 2:** Robson classification groups

Robson group	≤19 years (%)	20-34 years (%)	≥35 years (%)	Unknown age (%)	Total	p-value	OR (≥35 vs. 20-34)
0-2	0.33	0.26	0.28	0.44	86,377	<0.001	1.083
3-5	0.51	0.37	0.44	-	126,676	<0.001	1.196
6-7	1.83	1.42	1.64	0.44	476,561	<0.001	1.15
8-10	94.37	95.84	96.00	23.01	29,978,668	<0.001	01.02
Unknown	2.97	2.10	1.63	76.11	683,042	<0.001	0.78
Total	5,149,998	21,705,887	4,494,761	678,031	31,351,324		

For delivery mode (vaginal or cesarean), results showed that vaginal delivery rates were generally higher in Group 2 (20-34 years). Among women aged 20 to 34, the mean vaginal delivery rate was 41.84%, while for women aged 35 and above, the mean was 36.22%. The cesarean delivery rate in the 35 to 69 age group averaged 63.34%, with significant increases in the 35-54 age range (69.68%, 70.14%, 69.96%, and 68.09%) compared to 58% in the 20-34 age group. For the 5-minute APGAR results, the difference between the control group and the AMA group in scores < 7 was only 0.31% (2.36% - 2.05%). The analysis of over 31 million live births in Brazil (2012-2022) revealed significant differences in neonatal outcomes by maternal age. Compared to the reference group (20–34 years), adolescents (≤19 years) had the highest risk of low APGAR scores (<8 at 5 minutes), with an odds ratio of 1.29 (95% CI: 1.28–1.30, *p*<0.0001). Women of advanced maternal age (≥35 years) also showed an increased risk (OR=1.15, 95% CI: 1.14–1.16, *p*<0.0001), though less pronounced than adolescents. The reference group (20–34 years) demonstrated the most favorable outcomes, with only 2.05% of births presenting low APGAR scores. These findings highlight that both extremes of maternal age are associated with higher risks of neonatal distress, warranting targeted clinical attention (Table 3).

**Table 3:** APGAR Score at 5 Minutes: < 8 x 8-10. Control Group x AMA

Maternal Age	APGAR 5’ <8 (%)	APGAR 5’ 8–10 (%)	Unknown (%)	Total (n)	OR (95% CI)	p-value
≤19 years	2.63	94.37	2.97	31,351,324	1.29 (1.28–1.30)	<0.0001
20–34 years (Ref.)	2.05	95.84	2.10	21,705,887	1.00 (reference)	–
≥35 years (AMA)	2.36	96.00	1.63	4,494,761	1.15 (1.14–1.16)	<0.0001
Unknown	0.88	23.00	76.11	678	–	–

Regarding birth weight, most births occurred in the 2500 to 3999-gram range in both Group 1 (mean 22.81%) and Group 2 (mean 22.03%). However, women aged 35 and older exhibited a higher proportion of low birth weight (LBW) neonates, especially in the 1500 to 2499-gram range, compared to the control group. Among women aged 20 to 34, the mean LBW rate was 6.74%, while it was 12.48% in AMA women. For congenital anomalies, the prevalence was higher across all age ranges starting at 35 years compared to younger women. This study, analyzing 264,094 cases of congenital anomalies among 31 million births, demonstrated significant variation in prevalence according to maternal age (p<0.001). Compared to the reference group (20-34 years; 0.78%), women ≥35 years showed higher prevalence (1.16%) and increased risk of anomalies (OR=1.48; 95%CI:1.47-1.50), reinforcing the association between advanced maternal age and adverse outcomes. Adolescents (≤19 years) exhibited a modestly elevated risk (0.82%; OR=1.06; 95%CI:1.05-1.07). Regarding specific types of congenital anomalies, as classified by DATASus—such as spina bifida, central nervous system defects, cleft lip and palate, genitourinary tract anomalies, foot malformations, and musculoskeletal anomalies—proportions were higher in younger age groups. Musculoskeletal anomalies were notably different (27.14% in Group 2 and 18.99% in Group 1). Conversely, circulatory system anomalies (10.42% in Group 2, 14.61% in Group 1), chromosomal abnormalities (2.50% in Group 2, 12.60% in Group 1), and other anomalies (13.05% in Group 2, 14.94% in Group 1) were more prevalent in AMA pregnancies. No relevant differences were observed in the prevalence of anomalies such as esophageal, testicular, gastrointestinal, hip, or hemangioma/lymphoma malformations across age groups (Table 4).

**Table 4:** Occurrence of congenital malformations

Maternal Age	Yes (n)	No (n)	Total	Prevalence (%)	OR (95% CI)	p-value
≤19 years	42,081	5,107,908	5,149,989	0.82	1.06 (1.05-1.07)	<0.001
20–34 years (Ref.)	170,088	21,535,743	21,705,831	0.78	1.00 (Reference)	-
≥35 years (AMA)	51,921	4,442,828	4,494,749	1.16	1.48 (1.47-1.50)	<0.001
Unknown	4	674	678	0.59	-	-

## Discussion

According to Pinheiro et al.,^(4)^ as maternal age advances, the risk of preterm birth increases. This study aligns with our findings, which showed a general increase in the number of preterm births among AMA patients, particularly in the 50 to 54 age range (18.78%). Conversely, a study by Marques et al.^(7)^ found no relationship between maternal age and preterm births in the 35-40 age group, which does not align with our data. However, above 40 years of age, Marques et al.^(7)^ observed a relationship between AMA and gestational duration, consistent with the results we obtained from DATASus. It is also worth noting that, although the association between AMA and preterm births remains observable in some countries, this trend has been decreasing over the years.^(7,8)^ Therefore, reevaluating the relevance of maternal age in determining invasive procedures, such as cesarean delivery, in older pregnant women is valid. Additionally, it is important to consider that in low- and middle-income countries (LMIC), such as Brazil, these trends may be influenced by limited access to prenatal care and higher rates of unmanaged comorbidities, factors that can exacerbate adverse outcomes associated with AMA.

Regarding the number of fetuses per pregnancy based on maternal age, our results demonstrated that the occurrence of twin and triplet pregnancies is associated with increasing maternal age. These findings align with the statement by the UK National Collaborating Centre for Women's and Children's Health that the incidence of multiple births has increased in recent years, linked to rising maternal age at conception and changes in population demographics.^(9)^ Similarly, Marques et al.^(7)^ observed a significant increase in the use of assisted reproductive technologies among AMA women. Thus, the incidence of multiple pregnancies is rising, primarily due to delayed motherhood and the consequent widespread use of assisted reproductive techniques.^(7,9)^ It is inferred that the results in our study are partially explained by the growing demand for assisted reproduction by women aged 35 and older. Finally, it is important to highlight that absolute rates of pregnancy complications are higher in multiple pregnancies. Therefore, maternal age should be considered when assessing gestational risks, particularly in cases of multiple pregnancies, to provide more targeted care and interventions that lead to better outcomes.^(10,11)^

Pinheiro et al.^(4)^ reported that women with AMA have a higher likelihood of undergoing elective cesareans. This result was also observed in the DATASus data analysis for the Robson classification variable, revealing a predominance (28.96%) of AMA women in Group 5 (previous cesarean, single, cephalic fetus, >37 weeks). While this group also represents the majority of mothers aged 20 to 34 years (19.05%), these numbers indicate a significantly lower proportion compared to older women, suggesting that mothers of advanced age are more likely to have previous cesareans and to opt for or require additional cesareans. Additionally, summing Groups 1, 2, and 6 of the Robson classification, AMA mothers were nearly twice as prevalent in terms of nulliparity compared to younger mothers. These results may be explained by the greater difficulty in conceiving and maintaining a pregnancy as age advances. There is a clear correlation between advancing maternal age and reduced conception success, whether spontaneous or through in vitro fertilization. Both diminished ovarian reserve and oocyte quality contribute to this scenario. Reduced development to the blastocyst stage and/or chromosomal abnormalities are the main consequences. The influence of age on endometrial function may also significantly affect implantation rates, pregnancy rates, and overall female fertility. Endometrial aging observed in AMA pregnancies induces molecular, cellular, and histological changes that negatively impact endometrial receptivity and biology. This is associated with chronic conditions that can increase pro-inflammation and lead to endometrial tissue fibrosis, further compromising embryo attachment and pregnancy continuation.^(12,13)^

In terms of delivery mode, cesarean rates were higher on average among AMA women than among those aged 20 to 34. Our findings are consistent with two other studies on the same demographic segment, where cesarean rates in Group 1 were 60.05% and 60.3%.^(14,15)^ Reasons for the high cesarean rates include factors such as a greater prevalence of comorbidities or complications associated with AMA pregnancies, labor induction, and abnormal fetal positions. Many women opt for elective cesareans, especially primigravidae, often influenced by fear, which can be exacerbated in a highly anticipated pregnancy or after assisted reproductive treatments. Additionally, women attempting vaginal delivery after a previous cesarean may face increased risks of labor progression failure and uterine rupture.^(16)^

Regarding the APGAR score, while Pinheiro et al.^(4)^ found a correlation between APGAR <7 and maternal age ≥35 years, we did not observe such a correlation in our data.

According to table 3, there was no significant difference between the groups below 35 years and those 35 years or older. Our findings align with the study by Ratiu et al.,^(17)^ which also did not identify a clear association between higher maternal age and lower APGAR scores at 5 minutes. According to that study, a confounding bias may exist, as AMA predisposes women to other comorbidities, such as hypertension and diabetes. Thus, the association may be between maternal comorbidities and low APGAR scores, rather than directly with maternal age. A similar conclusion was drawn in the study by Razaz et al.,^(18)^ which found that maternal comorbidities during delivery are the primary causes of complications and low APGAR scores, rather than maternal age itself.^(17,18)^

When analyzing birth weight distribution by maternal age, our study highlighted significant differences between Groups 2 and 1 in terms of LBW, particularly in the 1500 to 2499-gram range. Similarly, the study by Wang et al.^(5)^ found that the risk of LBW for maternal age starting at 36 years increased by 13.3% per additional year of maternal age (OR = 1.133; 95% CI: 1.026–1.250). It is worth noting that the referenced study had a limited age range, excluding women over 40 years, unlike our study. Such differences underline the importance of conducting specific analyses in LMIC settings, where birth outcomes are further influenced by socioeconomic and healthcare disparities.

Regarding congenital anomalies and maternal age, beyond numerical chromosomal abnormalities, only circulatory system anomalies and "other malformations" showed higher rates in Group 1 compared to Group 2. Literature on this subject indicates a general U-shaped risk trend, where very young and older mothers have a higher risk of having children with anomalies, including non-chromosomal ones (NCAs). A study by Pethő et al.^(19)^ showed that the relative risk (RR) for NCAs between mothers aged 20-30 and those over 40, without coinciding chromosomal anomalies, was 1.25 (95% CI: 1.08–1.46), indicating a 25% higher risk of NCAs in older mothers when chromosomal anomalies were not present.^(19)^ Conversely, our data clearly show a significant prevalence of chromosomal anomalies in Group 1 (12.60%) compared to Group 2 (2.50%).

Although our study focuses on AMA as a variable of interest for different outcomes, some additional points warrant discussion. One of these concerns pregnancies in girls under 14 years, totaling 247,293 cases during the study period. According to Brazilian law, any sexual act involving a child or pre-adolescent under 14 is automatically considered rape, even if the victim claims consent. Moreover, pregnancies in this age group are high-risk and associated with specific biological, psychological, and social comorbidities, as well as maternal and infant mortality.^(20)^ Pregnancies between 14 and 19 years, while not necessarily criminal, are still classified as adolescent pregnancies by the World Health Organization, carrying distinct psychosocial characteristics compared to those of women of an appropriate reproductive age. For these reasons, we excluded these age groups from our analysis and discussion, although the tables include this data as part of the overall birth records during the study period.

Research on the impacts of AMA revealed significant findings in various gestational outcomes. A substantial increase in preterm births and multiple pregnancies was observed, driven, among other factors, by the rising use of assisted reproductive technologies among the study's target group. The data also indicate a higher prevalence of elective cesareans among AMA women, attributed to increased gestational risks associated with this age group, independent of comorbidities, as well as a higher frequency of comorbidities and multiple pregnancies. Furthermore, the association between AMA and increased risk of congenital anomalies, both chromosomal and non-chromosomal, highlights the urgent need for adaptive health policies. This is particularly relevant in LMICs like Brazil, where structural inequalities in healthcare access and maternal support services further compound the risks associated with advanced maternal age. In the Brazilian context, where maternal health policies require constant updating, our results emphasize the importance of a multidisciplinary and comprehensive approach to address diverse maternal realities. This includes offering specialized preconception care and implementing, disseminating, and enforcing guidelines for managing high-risk pregnancies, particularly those involving older women, across all prenatal care services, from major referral centers to small family health units.

## Conclusion

Advanced maternal age is associated with increased gestational and perinatal risks, including higher rates of prematurity, cesarean deliveries, low birth weight, and congenital anomalies. These findings highlight the need for individualized prenatal care and public health policies aimed at improving maternal and neonatal outcomes in this population.
